# Forest Vegetation of the Colombian Orinoquia: Characterization and Spatial Distribution Across Environmental Gradients

**DOI:** 10.3390/plants15111606

**Published:** 2026-05-24

**Authors:** Larry Niño, Orlando Rangel, Diego Giraldo-Cañas, Daniel Sánchez-Mata, Vladimir Minorta-Cely

**Affiliations:** 1 Natural Science Institute, National University of Colombia, Bogotá D.C. 111321, Colombia; lninoa@unal.edu.co (L.N.); jorangelc@unal.edu.co (O.R.); dagiraldoc@unal.edu.co (D.G.-C.); 2 Botany Unit, Faculty of Pharmacy, Complutense University of Madrid, 28040 Madrid, Spain; dsmata@ucm.es; 3 Department of Organismic and Evolutionary Biology (OEB), Harvard University Herbaria, Harvard University, Cambridge, MA 02138, USA; 4 Biology Program and Natural Sciences Services, Central University, Bogotá D.C. 111711, Colombia

**Keywords:** neotropical savannas, phytosociology, forests mapping, random forest, multi-sensor remote sensing, environmental gradients, Colombian orinoquia

## Abstract

Vegetation spatial heterogeneity is fundamental to biodiversity management and ecosystem service provision, yet detailed phytosociological mapping of forest vegetation remains largely unresolved in the Colombian Orinoquia. This study characterized the geographic distribution of forest vegetation through the integration of 178 field surveys, environmental complex variables defined by geomorphological and bioclimatic gradients, and multi-sensor satellite imagery combining Landsat-8 optical bands and Sentinel-1 dual-polarization data, processed within a Random Forest classification framework in Google Earth Engine. Classifications achieved overall accuracies between 0.910 and 0.975 and Kappa coefficients above 0.93, identifying 24 phytosociological alliances or geobotanical formations distributed across approximately 7,565,696 ha, representing 34.63% of the region. Forest cover ranges from 10.95% in the Floodplain to 55.22% in La Macarena, with the High Plain concentrating the greatest formation diversity. The spatial organization of forest vegetation is primarily governed by the geomorphological gradient—fluvial, denudational, and structural—and limiting bioclimatic factors, together with their associated edaphic−hydrological regimes, with anthropic transformation driven by cattle ranching and agricultural expansion constituting the principal threat to forest cover. These results advance beyond existing land cover surrogates, providing an empirically validated cartographic framework for biodiversity assessment, habitat modeling, and natural capital management in the Colombian Orinoquia.

## 1. Introduction

The spatial heterogeneity of vegetation, understood as the set of plants that establish spontaneously in a particular area [1], is fundamental to land-use planning and biodiversity management, given that primary productivity supports most of the ecological functions upon which the abundance and distribution of other life forms depend [2]. As a fundamental structural component of terrestrial ecosystems, vegetation regulates fluxes in biogeochemical cycles [3], influences climatic processes, the albedo effect, and the exchange of water and heat [4]; its patterns respond to environmental factors such as climate and topography, the physiological responses of the plant populations present, and the structural and functional characteristics of vegetation types [5]. In the Orinoquia, the geographic distribution, structure, and floristic composition of vegetation are influenced by environmental heterogeneity, particularly by physiographic and climatic variations [6,7,8,9,10], which is why understanding its diversity and distribution across different scales is essential for the provision of ecosystem services and the management of natural capital [11,12].

Environmental complexes, defined as the sum of interrelated physical factors that shape landscapes along gradients [5], influence biodiversity through factors that limit the eco-physiology of organisms, disturbances that affect environmental conditions, and resources assimilable by organisms [13,14]. While bioclimatic gradients are direct determinants of species physiology, topographic gradients show high correlation with the distribution of biota [15,16,17,18]; mechanistic models integrating spectral data with climatic factors have demonstrated strong performance in the spatial characterization of vegetation [19]. Although the temporal and spatial resolution of climatic data had previously constituted a limiting factor in the characterization of biodiversity at regional scales, modeling studies have overcome these challenges through static and probabilistic correlations between occurrence points and climatic means over extended periods [20,21], with bioclimatic indices serving as valuable tools for establishing thresholds associated with climatic periods that impact vegetation through water and thermal stress [22,23]. Within this framework, geomorphological factors, together with mesoclimatic patterns, constitute the main predictor variables of the geographic distribution of current vegetation along diverse environmental gradients [24,25,26,27,28,29,30].

Characterizing these gradients and their influence on vegetation at regional scales requires spatially continuous data that fieldwork alone cannot provide. Optical remote sensors allow territories to be described by overcoming the spatial, temporal, and economic limitations of fieldwork [31]; their use in the elaboration of vegetation maps is based on the reflectivity and emissivity of plants captured from space, which allows the differentiation of elements on the Earth’s surface [32] and the identification of functional characteristics of dominant plants as predictors of physiological adaptations to environmental conditions [33,34,35], enabling the mapping of floristic assemblages with unique taxonomic compositions in heterogeneous landscapes [36,37]. Complementarily, Synthetic Aperture Radar (SAR) imagery provides information on the physical and dielectric properties of the natural environment [38,39], and its integration with optical imagery allows the enhancement of geobotanical landscape features and overcomes limitations in the statistical differentiation based on spectral variability in digital classifications [40,41,42,43]. Given the challenges posed by the processing of large volumes of satellite data at extensive scales, platforms such as Google Earth Engine (GEE) offer a solution by providing automated parallel computing with access to petabytes of planetary-scale remote sensing information, overcoming the computational capacity and storage limitations of traditional cartographic techniques [44,45,46,47,48].

The characterization of the Earth’s surface using remote sensors has evolved from the visual comparison of images toward automated digital classification methods, grouped into unsupervised categories, which cluster pixels by minimum distance, and supervised categories, which assign classes based on training areas with a high degree of certainty [49,50,51]. In the Colombian Orinoquia, although thematic cartography has been predominantly based on visual interpretations, parametric models such as maximum likelihood have been implemented [52,53,54]; however, Machine Learning techniques, in particular Random Forest, have gained special attention for their high accuracy, rapid processing, and non-parametric nature, which facilitates the classification of data that do not follow a normal distribution and of multidimensional images from diverse sources [55,56,57,58].

Biodiversity conservation in Colombia relies heavily on surrogates such as land cover and land-use maps; however, these tools present limitations when their correspondence with actual biodiversity distribution is low [59,60]. More detailed thematic cartography has demonstrated greater effectiveness in resolving environmental conflicts and supporting biodiversity management [36,61]. The use of multi-sensor approaches opens new technological pathways and helps overcome the inherent constraints of individual sensors [48]. Combining field surveys with satellite imagery is key to closing the gap between remote sensing and ecology, enabling scalable, continuous, and detailed biodiversity assessments [34,62,63]. Against this backdrop, the present study advances the characterization of vegetation diversity and distribution across the Colombian Orinoquia by integrating phytosociological field data, geomorphological and bioclimatic gradients, and multi-sensor satellite imagery—specifically Landsat-8 optical bands combined with Sentinel-1 dual-polarization data. The overarching goal is to strengthen the link between remote sensing and ecological knowledge, ultimately contributing to more robust biodiversity assessment and natural capital management at regional scales.

## 2. Results

Random Forest algorithms were applied to stacked spatial data to assign pixels to discrete units corresponding to the phytosociological alliances or formations previously delineated. Decision tree parameterization varied by landscape unit, with optimal values of 60 for the Foothills and 110 for the High Plain, yielding overall accuracies of 0.975 and 0.910, respectively—indicating that 91–97% of classified pixels matched their reference categories. Evaluation through confusion matrices considered diagonal concordance, error distribution patterns, and the non-random nature of pixel assignments. Peak omission (OE) and commission (CE) errors reached 16.7% and 13.2%, both concentrated in the High Plain. The Kappa coefficient, which quantifies the divergence between automated and random classifications [64,65], exceeded 0.93 across all units, reflecting a minimal likelihood of chance-driven assignments. Relative to visual interpretation approaches, this digital methodology enhances consistency and reproducibility by reducing subjective bias [61,66]. At the alliance or formation level, omission and commission errors varied inversely with spatial representativeness: classes occupying less than 0.20% of the regional area—such as *Coccolobo caracasanae-Tapiriretion guianensis*, *Phenakospermo guyannensis-Attaleetion maripae*, and *Protio heptaphylli-Jacarandion obtusifoliae*—exhibited the highest omission errors (16.3–16.7%), reflecting the reduced availability of training samples for underrepresented classes in Random Forest models. Conversely, six alliances with comparatively higher spatial coverage—including *Attaleo maripae-Iryantherion laevis*, *Alchorneo triplinerviae-Maurition flexuosae*, *Oenocarpo minoris-Attaleion maripae*, and *Chamaedoreo pinnatifrondis-Sloaneion brevispinae*—showed commission errors exceeding their omission counterparts, consistent with the tendency of overrepresented classes to attract misclassified pixels from spectrally similar or structurally overlapping formations (Table 1). Together, the overall accuracies and Kappa values substantiate the reliability of the resulting classifications as a foundation for interpreting vegetation distribution patterns at the regional scale.

The following sections describe the geographic distribution of the 24 phytosociological alliances or geobotanical formations identified across the four subregions of the Colombian Orinoquia. Analyses of the current vegetation distribution reveal that 73.74% of its territory retains a natural vegetation cover. Of this area, 34.63% is occupied by 24 forest formations. The data presented in Table 1 correspond to 24 phytosociological alliances or geobotanical formations distributed across the Colombian Orinoquia, with a total area of approximately 7,565,696 ha. The alliance with the greatest representation is *Attaleo maripae–Iryantherion laevis* with 3,478,544 ha (14.89%), which distinguishes it markedly from the remaining units and suggests its dominant role in shaping the forest landscape of the region. It is followed in extent by *Spondiado mombinis–Viticion orinocensis* (1.82%), *Chamaedoreo pinnatifrondis–Sloaneion brevispinae* (1.45%), and *Bowdichio virgilioidis–Curatellion americanae* (2.32%), while most of the remaining alliances occupy between 0.62% and 1.33% of the total area. In contrast, the units with the least representation are *Phenakospermo guyannensis–Attaleetion maripae* (0.03%), *Protio heptaphylli–Jacarandion obtusifoliae* (0.04%), and *Coccolobo caracasanae–Tapiriretion guianensis* (0.09%), whose limited extent may indicate particular disturbance, overexploitation, or environmental conditions that restrict their distribution. Overall, the distribution of alliances reflects a marked asymmetry in territorial occupation, where a single unit concentrates nearly 15% of the total surface area, while the majority of formations are distributed in comparatively smaller and relatively equitable extents—a pattern that highlights the physiognomic and floristic heterogeneity characteristic of the forests of the Colombian Orinoquia.

### 2.1. Foothills Forests

**Figure 1:**
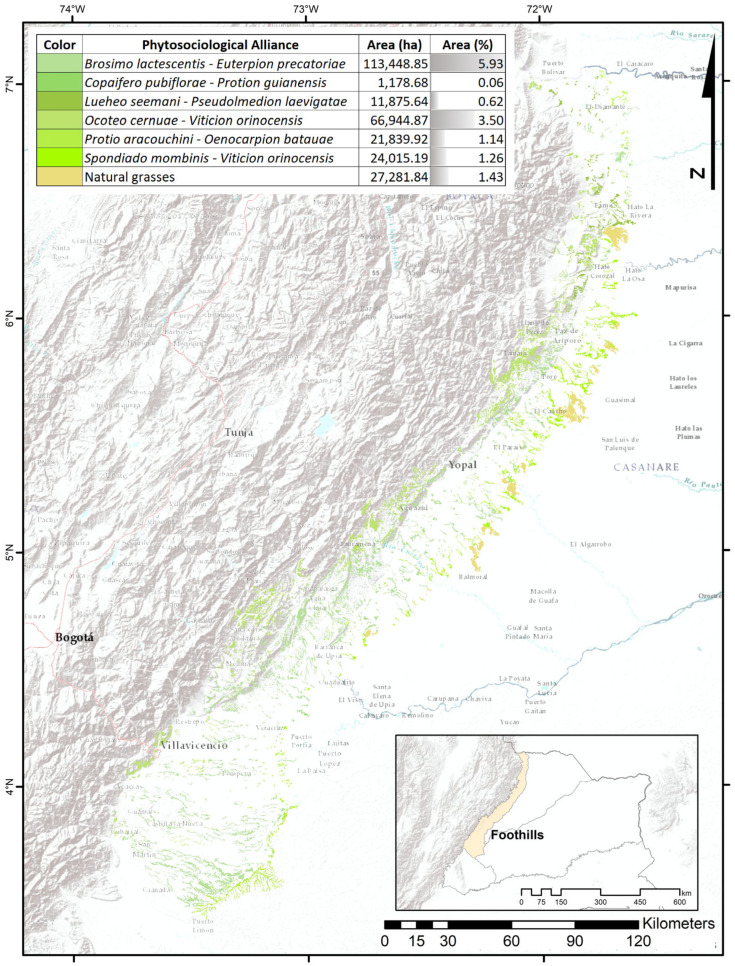
Spatial distribution and area of forests phytosociological alliances in the Foothills of the Colombian Orinoquia.

Natural forest vegetation represents only 12.51% of its territory (Figure 1), evidencing that 86.06% of natural cover has been transformed, primarily by the expansion of cattle ranching and monocultures such as oil palm and rice [67]. Six forest vegetation formations are recognized in this subregion, with a predominance of *Brosimo lactescentis–Euterpion precatoriae*, located in the fluvial environments of the main rivers, followed by *Ocoteo cernuae–Viticion orinocensis*, distributed toward the higher-elevation margin of the Foothills bordering the Eastern Cordillera, frequently occurring in structural geomorphological environments [57]. Natural forest remnants persist on steep terrain whose slope and edaphic characteristics are not conducive to agricultural activities; however, forests that have lost their original structure due to human intervention are maintained on these same terrains under continuous selective logging and, in more advanced stages of degradation, consist primarily of shrublands located on low-gradient terrain within the fluvial environment, where selective felling gives way to generalized clearing as a precursor to the establishment of agricultural activities [68].

### 2.2. La Macarena Forests

**Figure 2:**
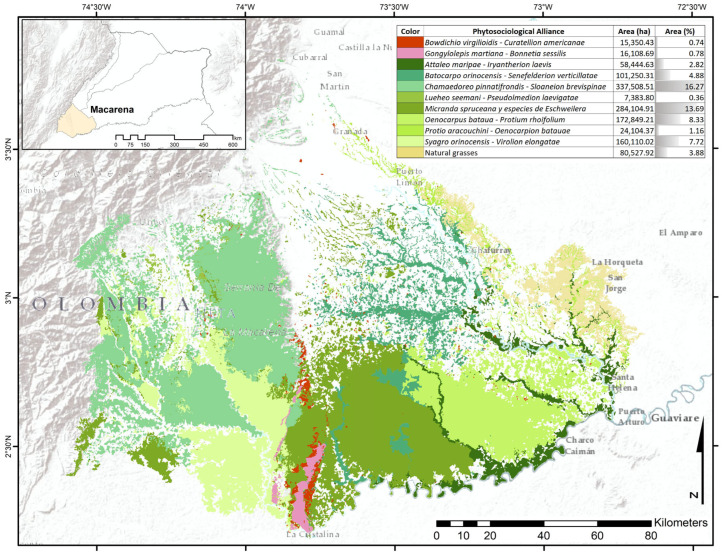
Spatial distribution and area of forests phytosociological alliances in La Macarena of the Colombian Orinoquia.

Natural forest vegetation represents 55.22% of its territory; however, 39.38% of natural cover has been transformed, primarily by the expansion of pastures for extensive cattle ranching, the enlargement of the agricultural frontier, uncontrolled burning, and human settlements—processes that advance preferentially through non-flooded forest areas with well-drained, less acidic soils [69,70,71]. Eight forest vegetation formations were identified in this subregion, with a predominance of *Chamaedoreo pinnatifrondis–Sloaneion brevispinae*, rooted predominantly in the structural and denudational environments of the massif and the cordilleran foothills, followed by *Micranda spruceana* and species of *Eschweilera*, distributed over denudational hills north of the Guayabero River, and *Oenocarpus bataua–Protium rhoifolium*, located likewise over denudational hills between the Central River and the mouth of the Ariari River. Complementarily, *Syagro orinocensis–Virolion elongatae* is distributed across fluvial and denudational environments on the western slope of the massif and on terraces south of the Guayabero River, while the low forest of *Bowdichio virgilioidis–Curatellion americanae* occupies the low-gradient structural environment, and the shrubland of *Gongylolepis martiana–Bonnetia sessilis* is restricted to structural hillsides and slopes of the massif with frequent rocky outcrops (Figure 2).

### 2.3. Floodplain Forests

**Figure 3:**
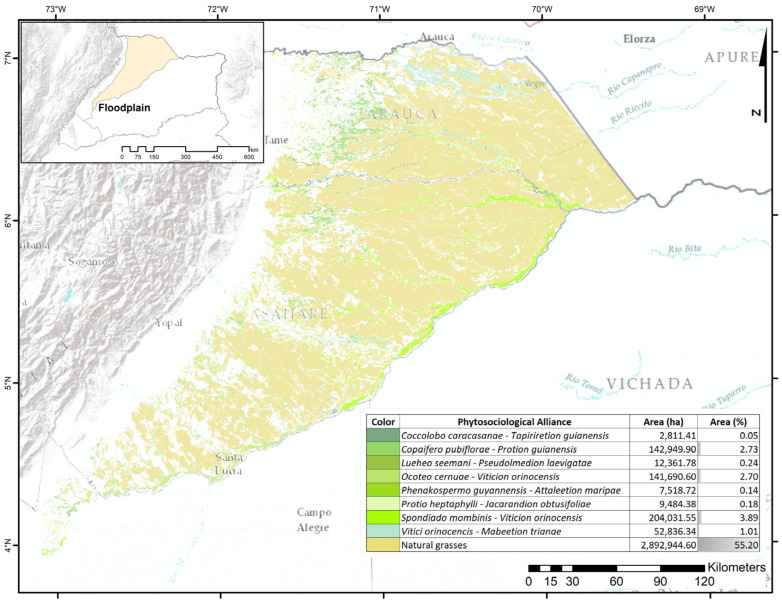
Spatial distribution and area of forests phytosociological alliances in the Floodplain of the Colombian Orinoquia.

Natural forest vegetation represents only 10.95% of its territory, while human activities have transformed 33.86% of natural cover, with agricultural and livestock modifications located primarily in lowlands, floodplain banks, and river levees of the Lipa, Ele, and Cravo Norte rivers and their tributaries, where acidity levels and aluminum concentrations are lower than on the terraces [72]. Eight forest formations were identified in this subregion, all associated with floodplains, with a predominance of *Spondiado mombinis–Viticion orinocensis*, located primarily on the floodplains associated with the tributaries of the Meta River in the central and northern sections of the eastern flank, followed by *Copaifero pubiflorae–Protion guianensis* and *Ocoteo cernuae–Viticion orinocensis*, distributed over floodplains associated with denudational plains located toward the northwest and over the tributaries of the Meta River to the south, respectively. The formations with lesser spatial representation include *Vitici orinocencis–Mabeetion trianae* on the floodplains of the Arauca River tributaries to the north; *Lueheo seemani–Pseudolmedion laevigatae* on the floodplains of the Lipa and Casanare rivers adjacent to denudational plains toward the northwest; *Protio heptaphylli–Jacarandion obtusifoliae* on the floodplains of the Cinaruco River associated with denudational and aeolian environments; *Phenakospermo guyannensis–Attaleetion maripae* in the northeastern extreme where the aeolian environment prevails; and *Coccolobo caracasanae–Tapiriretion guianensis* on plains adjacent to loess sheets near the Meta River (Figure 3).

### 2.4. High Plain Forests

**Figure 4:**
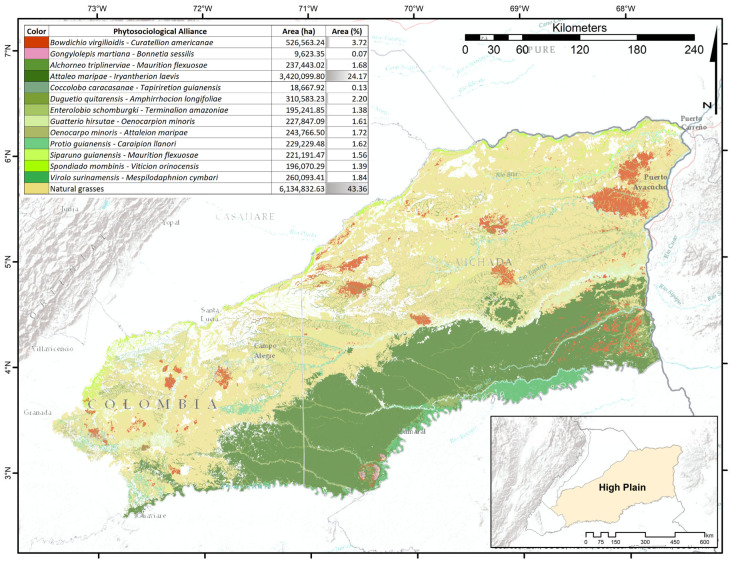
Spatial distribution and area of forests phytosociological alliances in the High Plain of the Colombian Orinoquia.

Natural forest vegetation represents 39.30% of its territory (Figure 4), making this the subregion with the greatest diversity of forest formations, totaling 11, where anthropic activity has transformed 13.55% of natural covers, particularly within the fluvial environment, where chemical reduction improves soil fertility properties, favoring the establishment of agricultural and livestock activities in originally forested areas [68]. *Attaleo maripae–Iryantherion laevis* predominates extensively, its distribution encompassing the denudational environment bounded to the east by the Siare River, to the west by the Orinoco River, to the north by the highly dissected hills associated with the Vichada River, and to the south by the fluvial environment of the Guaviare River. It is followed in spatial representation by *Duguetio quitarensis–Amphirrhocion longifoliae*, associated with the fluvial environment adjacent to the areas where *Attaleo maripae–Iryantherion laevis* predominates, whose geomorphological units are influenced by the main tributaries of the Guaviare and Orinoco rivers.

The formations of intermediate representation in the High Plain are predominantly associated with narrow fluvial units and denudational environments. *Virolo surinamensis–Mespilodaphnion cymbari* is located in the fluvial environment of the Guaviare River and its tributaries Siare, Iteviare, and Uva; *Oenocarpo minoris–Attaleion maripae* occupies narrow fluvial units adjacent to residual hilly plains of the Tuparrito stream and the Vichada and Tuparro rivers, as well as peneplains and hills near the Melúa, Muco, Elvita, and Tomo rivers and the upper basin of the Tuparro River; *Alchorneo triplinerviae–Maurition flexuosae* is distributed over dissected hills in the upper basins of the Melúa and Vichada rivers, highly dissected hills near the Cumaral, Cumachabo, and Rubiales streams and the Manacacías, Planas, Tillava, and Guarrojo rivers, peneplains associated with the Muco and Tomo rivers, and residual hilly plains near the Bita and Tomo rivers; *Protio guianensis–Caraipion llanori* occupies fluvial units of the denudational environment in the vicinity of the Ovejas stream, hills in the upper basin of the Manacacías River, highly dissected hills between the Meta and Yucao rivers, and floodplains associated with the Tomo River; *Guatterio hirsutae–Oenocarpion minoris* is distributed over fluvial units of the Manacacías and Vichada rivers; and *Siparuno guianensis–Maurition flexuosae* occupies narrow fluvial units over hills influenced by the Cuamaral, Melúa, Yucao, and Manacacías rivers and the middle basin of the Planas River, the peneplain of the upper basin of the Muco River, hilly plains in the middle basins of the Tomo and Tuparro rivers, and the tributaries of the Bita River and the Liqui stream.

The formations with lesser representation in the High Plain include: *Spondiado mombinis–Viticion orinocensis*, associated with the fluvial units of the Meta, Juriepe, and Bita rivers; *Enterolobio schomburgki–Terminalion amazoniae*, occurring in the fluvial environment of the Orinoco, Tuparro, Tomo, Mesetas, and Guio rivers and their minor tributaries; *Coccolobo caracasanae–Tapiriretion guianensis*, in the fluvial environment of the Meta River; the low forests of *Bowdichio virgilioidis–Curatellion americanae*, distributed primarily over the inselbergs near the Mapiripana rapids on the Guaviare River, fill-spill plains, and hilly plains along the eastern margin of the Orinoco River, and the Matavení, Mono, and Fruta streams in the southwestern extreme of the High Plain; and the shrublands of *Gongylolepis martiana–Bonnetia sessilis*, restricted to the inselbergs near the Mapiripana rapids on the Guaviare River.

Taken together, the results reveal marked contrasts in forest cover, formation diversity, and degree of anthropic transformation across the four subregions of the Colombian Orinoquia. Forest cover ranges from 10.95% in the Floodplain and 12.51% in the Foothills—the most heavily transformed subregions, where cattle ranching, monocultures, and agricultural expansion have altered the majority of natural covers—to 39.30% in the High Plain and 55.22% in La Macarena, where anthropic transformation remains comparatively lower at 13.55% and 39.38%, respectively. Formation diversity follows a similar gradient, increasing from six formations in the Foothills to eleven in the High Plain, which concentrates the greatest phytosociological complexity of the region. Across all subregions, the distribution of formations is consistently structured by geomorphological environment—fluvial, denudational, structural, and aeolian units—reflecting the primacy of physiographic factors as spatial determinants of forest composition. The pronounced asymmetry in territorial occupation, where *Attaleo maripae–Iryantherion laevis* alone accounts for nearly 15% of the total forested area, contrasts with the relatively equitable distribution of the remaining alliances and underscores the physiognomic and floristic heterogeneity that characterizes the forests of the Colombian Orinoquia.

## 3. Discussion

This study characterized the geographic distribution of current vegetation in the Colombian Orinoquia through an empirical−statistical modeling approach, based on the relationship between stacked multidimensional image data—comprising optical and SAR-derived spectral variables, geomorphological units, and bioclimatic factors as predictors—and phytosociological forest alliances as the response variable. The integration of multi-source and multi-thematic data was key to discriminating vegetation types, providing complementary information on the geometry and texture of vegetation units and reducing the uncertainty associated with structural and spectral similarities [49,66,73,74]. The validated classifications identified 24 phytosociological alliances or geobotanical formations distributed across approximately 7,565,696 ha of forest cover, representing 34.63% of the region. The results reveal marked contrasts in forest cover, formation diversity, and degree of anthropic transformation across the four subregions, with the High Plain concentrating the greatest phytosociological complexity and *Attaleo maripae–Iryantherion laevis* emerging as the dominant formation at the regional scale. Collectively, these findings demonstrate that the spatial organization of forest vegetation in the Colombian Orinoquia is primarily structured by the environmental complexes and their associated edaphic−hydrological regimes, with anthropic transformation constituting the principal driver of forest cover reduction in the most accessible subregions.

The spatial discrimination of forests presents broad room for improvement through the integration of data from diverse sensors and thematic sources, such as those considered here; moreover, expanding the spectral and thematic resolution through multidimensional imagery could further improve overall accuracy and reduce variance in classifications [75,76]. Classification depends on the quality of training samples [77,78], which are derived from field surveys and reflect the spectral and landscape variability of forests. Likewise, balanced samples across thematic classes, incorporated through K-means pre-classification, increase accuracy and reduce commission and omission errors [79,80]. Although the initial K-means classification for defining training areas is useful for establishing sample representativeness, future studies could consider stratified sampling designs for selecting balanced training data based on prior thematic cartography [78,81]. In this study, the procedure was applied strictly as a spatial delineation tool whose output did not determine class labels—defined exclusively by field-based phytosociological criteria—ensuring that the classifier discriminates between ecologically meaningful units rather than algorithmically generated groupings. The spectral coherence of training pixels is therefore an inherent property of the delineation strategy, not a source of systematic bias, and is acknowledged as a boundary condition of the approach. Remaining limitations concern the potential underrepresentation of intra-alliance spectral variability, particularly in structurally complex vegetation units characteristic of the Orinoquia region. Specifically, the enforcement of spectral homogeneity during training area delineation may restrict the inclusion of transitional or mixed pixels that, while phytosociologically valid, deviate from the dominant spectral signature of each alliance. This could affect model sensitivity in ecotonal zones and should be considered when interpreting classification uncertainty in heterogeneous landscapes. Additionally, future studies could explore other capabilities of Random Forest, as this technique allows the variables included to be ranked according to their relevance in discriminating vegetation types, which could prove useful for incorporating new variables or refining those considered in the present study [56,82].

Regarding the optical sensor, the use of the annual per-pixel median could reduce the phenological influence associated with seasonal alternation, thereby improving classification precision [78]. However, persistent cloud cover over areas of the Foothills represents the primary limitation of the optical sensor, which may be mitigated by implementing collections with greater scene availability or by extending the image acquisition period used to construct the mosaic, although this entails a reduction in temporal resolution.

The accuracy levels achieved by the classifications, together with the integration of phytosociological field data and environmental complex variables, provide a sufficiently robust spatial foundation for ecological interpretation at the subregional scale. The distributional patterns emerging from this framework reveal the principal drivers structuring forest vegetation across the Colombian Orinoquia.

The results show that the best-preserved forest remnants are concentrated on steep terrain, dissected hills, or areas distant from the population centers of the Foothills. These territories constitute persistent refugia, where low agricultural suitability has acted as a factor of passive protection [83,84]. This observation supports the theory of remnant conservation in anthropized landscapes, where biodiversity congregates in areas marginal to production [85,86,87]. However, even these refugia are not exempt from impact. The sequence described—encompassing forests with altered structure due to selective logging, forest-shrubland mosaics, and those embedded in matrices of pastures or agricultural areas—constitutes a clear gradient of ecological degradation. This continuum may be interpreted as a regressive succession driven by the intensity and frequency of human intervention [87,88]. Thus, the shrubland strips surrounding degraded forests should not be interpreted a priori as merely ruderal vegetation series, as they may represent stages of various successional trajectories—early and intermediate—maintained by recurrent and constant disturbances [89,90].

The identification of clearly dominant alliances that constitute key catenary and defining elements in the region—such as *Attaleo maripae–Iryantherion laevis* in the Vichada–Guaviare interfluve and *Brosimo lactescentis–Euterpion precatoriae* in the Meta plains—suggests that current ecological conditions favor their establishment as climax elements in those areas, under the prevailing edaphic−hydrological regimes of their respective physiographic units. The spatial segregation of phytocoenoses is demonstrably non-random: alliances such as *Spondiado mombinis–Viticion orinocensis* and *Duguetio quitarensis–Amphirrhocion longifoliae* are intimately linked to floodplains and active fluvial environments, reflecting the broad and dynamic alluvial processes of the region. Conversely, alliances such as *Ocoteo cernuae–Viticion orinocensis* and *Chamaedoreo pinnatifrondis–Sloaneion brevispinae* are restricted to denudational and structural environments of slopes and massifs, reflecting a clear association with radically distinct soil and drainage conditions. It is therefore suggested that drainage networks exert a significant influence on the phytosociological arrangement of forests in the region. These geomorphological factors confirm that, together with mesoclimatic conditions, the environmental gradient present in each physiographic unit constitutes the primary modulator of the considerable beta diversity observed [91,92,93,94].

The patterns documented here are consistent with previous studies that highlight the Orinoquia as a region of transition and high beta heterogeneity [6,95,96,97,98]. The identification of alliances such as *Protio guianensis–Caraipion llanori* and *Alchorneo triplinerviae–Maurition flexuosae* in highly localized environments suggests that the phytosociological catalogue of Neotropical savannas may be substantially broader than currently recognized, and that units previously described in more general terms could be refined as spatially explicit mapping approaches are applied at finer scales [99,100,101]. However, confirming this interpretation requires caution, as the absence of long-term succession data prevents distinguishing whether these assemblages represent stable, climatically determined alliances or transitional states within broader successional trajectories. These findings advance the classical understanding that emphasized the flooding gradient primarily; the present results demonstrate that the geomorphological gradient—fluvial, denudational, and structural—constitutes an organizing axis of equal or greater importance for explaining not only the composition but also the ecogeographical extent of phytocoenoses at the subregional scale [92].

The present study contributes a spatially explicit and phytosociologically grounded characterization of forest vegetation in the Colombian Orinoquia, advancing beyond previous land cover approaches by resolving 24 alliances or geobotanical formations whose distribution is governed by the interplay of geomorphological and bioclimatic factors. Understanding vegetation patterns in megadiverse countries is critical for addressing global conservation challenges, providing an essential foundation for wildlife habitat studies [102], species and community distribution modeling [59,103,104], identification of high conservation value areas [36,105], assessment of threats and disturbance effects on biodiversity [33,106], protection and sustainable management of forest resources [74], and the biophysical estimation of environmental supply in territories, as well as the guidance of restoration, mitigation, and ecological compensation processes based on reliable information on dominant plant species [1]. These results therefore provide an empirically validated cartographic framework that can directly inform biodiversity assessments, ecosystem service valuation, and natural capital management at the regional scale—needs that current land cover surrogates used in Colombian conservation planning have been insufficient to address.

## 4. Materials and Methods

### 4.1. Study Area

The Colombian-Venezuelan Llanos constitute the second largest expanse of Neotropical savannas (≈532,000 km^2^) [107,108]. This region, of notable ecological complexity and floristic diversity, extends in Colombia from the Foothills of the Eastern Cordillera to the Orinoco River, encompassing 233,546 km^2^ [93]. Its geographic boundaries are demarcated by the Arauca River (north), the Orinoco River (east), the Guaviare River (south), and the Guayabero River basin (west) [109]. From a geomorphological perspective, two principal units are distinguished: the well-drained Orinoquia (east of the Meta River), characterized by dissected high plains and hills; and the poorly drained Orinoquía (west of the Meta River), dominated by alluvial and aeolian plains. Likewise, four physiographic units are recognized: the Foothills, the Flood/Aeolian Plain, the High Plain, and La Macarena [6,9,57,110].

### 4.2. Physiographic Setting

**Figure 5:**
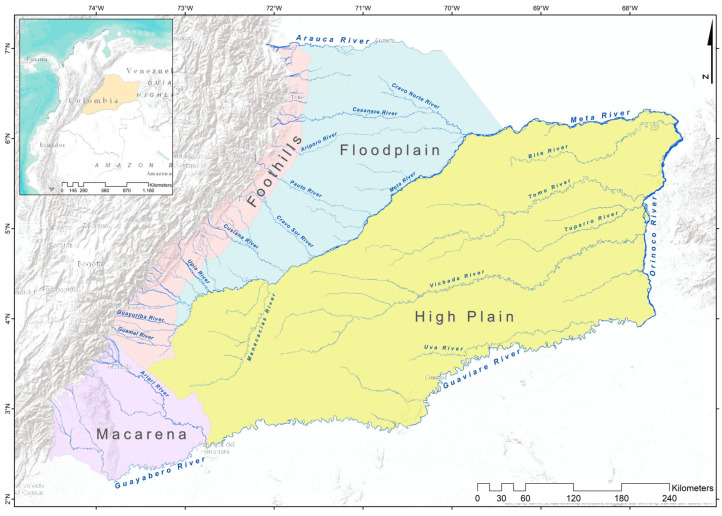
Spatial context of the study area, including physiographic units and the major drainage systems.

The physiographic delimitation was based on the hydrographic zoning of Colombia [111] and the NASA SRTM v.3 digital elevation model (30 m) [112] available in Google Earth Engine. The Foothills were delimited on their eastern and western flanks using altitudinal thresholds defined according to the hydrographic basins present. South of the Meta River, in the Guamal River basin, an upper threshold of 575 m and a lower threshold of 350 m were established. North of the Meta River, altitudinal ranges were differentiated by basin: 675–200 m for the Meta and Pauto rivers, 600–200 m for the Casanare River, 425–200 m for the Cravo Sur River, and 375–175 m for the Arauca River. At its northern extreme, the boundary corresponds to the Arauca River, while to the south it is defined by the Ariari River basin. La Macarena was delimited to the west by altitudinal thresholds of 575 m in the Guayabero River basin and 775 m in the Guaviare River basin; to the north and east by the boundaries of the Ariari River basin; and to the south by the Guayabero River and the limits of its basin. The Floodplain was bounded to the west by the Foothills, to the north by the Arauca River, to the east by the international border with Venezuela, and to the south by the Meta River. The High Plain, in turn, was delimited to the west by the Foothills and La Macarena, to the north by the Meta River, to the east by the Orinoco River, and to the south by the Guaviare River (Figure 5).

### 4.3. Modelling Framework and Data Training

The analysis was based on an empirical−statistical model designed to quantify the relationship between predefined phytosociological units, used as the response variable, and a set of bioclimatic predictors derived from WorldClim [113], as well as geomorphological predictors [93] and spectral predictors obtained from Landsat-8 and Sentinel-1 imagery. Unsupervised (K-means) and supervised (Random Forest) classification algorithms were applied to model the distribution patterns. The former defined training areas, and the latter generated the spatial classification.

Training areas were defined using 178 forest vegetation surveys conducted in the Colombian Orinoquía; the geographic location and main attributes of these vegetation surveys are available in [114], and their syntaxonomic classification was previously established by [115]. The phytosociological classification comprises the following syntaxonomic units: Class *Maquiro coriaceae–Copaiferetea pubiflorae*, encompassing the following orders: *Brosimo lactescentis–Oenocarpetalia minoris* (three alliances), *Iryanthero laevis–Oenocarpetalia batauae* (two alliances), *Alchorneo discoloris–Protietalia llanori* (five alliances), *Alibertio edulis–Mabeetalia trianae* (two alliances), and *Ocoteo bofo–Mabeetalia trianae* (three alliances). Class *Brosimo lactescentis–Eschweileretea subglandulosae*, including the orders: *Mabeo nitidae–Mespilodaphnetalia cymbari* (two alliances), and *Phenakospermo guianensis–Minquartietalia guianensis* (one alliance). Class *Jacarando copaiae–Luehetea seemani*, comprising the order: *Pouterio stipitatae–Terminalietalia amazoniae* (two alliances).

### 4.4. Data Collection and Processing

The detailed procedures for the acquisition and processing of input data are available in [57,93,94].

*Geomorphology*. The geomorphological model integrated two L-band SAR polarizations and five parameters considered fundamental terrain descriptors [116,117]: (i) HH/HV polarizations obtained from the ALOS PALSAR 2022 annual mosaic (Global PALSAR-2/PALSAR collection, available in GEE), to which speckle correction was applied using a 50 m focal mean filter [76,118]; (ii) the NASA SRTM v.3 DEM, from which cartographic layers of slope, aspect, and convexity were derived; (iii) slope, expressed as a percentage of inclination; (iv) aspect, defined as the azimuthal orientation of the slope in degrees relative to north; (v) convexity, calculated from the relative elevation of a seven-pixel moving window with respect to its neighborhood, classified as concave (elevation lower than surroundings), convex (higher), or flat (similar); and (vi) surface roughness, estimated using the local variance of slope within seven-pixel moving windows.

*Adjusted Potential Evapotranspiration.* The adjusted potential evapotranspiration cartographic layer describes the transfer of liquid water to the atmosphere in the form of vapor, integrating both evaporation from open surfaces and plant transpiration [119]. At the regional scale, it constitutes an indicator of climatically relevant aspects such as water balance, vegetation productivity, and biodiversity [4,120]. Its calculation followed the Thornthwaite method [121] in which the uncorrected mean monthly potential evapotranspiration (*ETP_na_*) is expressed as:

$${ETP}_{na\;}=16\;{\left(\frac{10\;T_{mm}}{I_{ca}}\right)}^{a}$$

where *T_mm_* is the mean monthly temperature (°C), *I_ca_* is the annual heat index—a measure of accumulated thermal energy—obtained by summing the twelve-monthly heat indices (*i_j_*):

$$I_{ca}=\;\sum_{j=1}^{12}i_{j},\;\;i_{j}=\;{\left(\frac{T_{mmj}}{5}\right)}^{1.514}$$

The exponent *a*, which functions as an estimator of atmospheric moisture retention capacity, is derived from *I_ca_* as:

$$a=\;\left(0.000000675\right){I_{ca}}^{3}-\;\left(0.0000771\right){I_{ca}}^{2}+\;\left(0.0000771\right)I_{ca}+\;0.49239$$

At regional and local scales, topography influences the distribution of solar radiation and, consequently, evapotranspiration. To obtain an adjusted estimate, a correction was applied that accounts for the number of days per month and the daily sunshine hours as a function of latitude and topography. This correction is expressed through the monthly illumination index *L_i_*:

$$L_{i}=\;\left(\frac{D_{i}}{30}\right)\;\times \;\left(\frac{H_{i}}{12}\right)$$

where *D_i_* is the number of days and *H_i_* the sunshine hours in month *i*, calculated using the GRASS r.sun.insoltime algorithm [122] based on the NASA SRTM v.3 DEM at 30 m resolution available in GEE. The adjusted mean monthly potential evapotranspiration (*ETP_a_*) is then obtained as:

$${ETP}_{a}=\;{ETP}_{na}\;\times L$$

Finally, the adjusted annual potential evapotranspiration was obtained by summing the twelve corrected monthly values.

*Ombrothermic index (annual and dry quarter)*. These indices establish a direct relationship between precipitation and temperature, providing an estimate of water availability as limited by evapotranspiration losses or by the reduction in rainfall efficiency with increasing temperature [23]. The annual ombrothermic index (*I_o_*) was calculated as:

$$I_{o}=\;\left(\frac{P_{pa}}{T_{pa}}\right)10$$

where *P_pa_* is the annual positive precipitation—defined as the sum of monthly precipitation (mm) for months in which mean monthly temperature exceeds 0 °C—and *T_pa_* is the annual positive temperature, defined as the sum of mean monthly temperatures above 0 °C. The ombrothermic index of the dry quarter (*I_od_*_3_) relates precipitation and temperature during the driest three-month period of the year and was computed as:

$$I_{od3}=\;\left(\frac{P_{ps}}{T_{ps}}\right)10$$

where *P_ps_* is the positive precipitation of the dry quarter and *T_ps_* is the positive temperature of the dry quarter, both restricted to months with mean temperatures above 0 °C.

*Thermicity index*. This index weights the intensity of extreme temperatures as a limiting factor for vegetation development [123]. Its calculation combined the mean annual temperature (*T_ma_*), the mean of minimum temperatures of the coldest month (*T_min_*), and the mean of maximum temperatures of the coldest month (*T_max_*), following:

$$I_{t}=\;\left(T_{ma}+\;T_{min}+\;T_{max}\right)10$$

*Sentinel-1 Mosaic*. This mosaic was generated from the dual-polarization C-band SAR GRD collection, preprocessed, calibrated, and orthorectified using the Sentinel-1 Toolbox, including ground range detection, thermal noise removal, radiometric calibration, topographic correction with the SRTM (30 m), and conversion to logarithmic decibel units [124]. The processing of 2022 imagery in GEE comprised: (i) selection of the interferometric wide swath mode; (ii) filtering by ascending, descending, or both orbital directions depending on topography; (iii) filtering by VV and VH polarizations; (iv) selection of data at 10 m spatial resolution; (v) clipping to the area of interest loaded as an Asset in GEE; (vi) alternating selection of VV and VH bands; (vii) temporal filtering for the full year; (viii) mosaic composition from both bands; and (ix) application of a 50 m circular focal mean filter to reduce speckle noise [94].

*Landsat-8 Mosaic*. This mosaic was generated from 2022 imagery of the USGS Landsat 8 Surface Reflectance Tier 1 collection, which includes bands from the visible spectrum (RGB), near-infrared (NIR), and shortwave infrared (SWIR), useful for vegetation differentiation [125,126,127]. These data are atmospherically and thermally corrected using the OLI/TIRS sensor and the LaSRC software [128]. Additionally, the Normalized Difference Vegetation Index (NDVI), calculated from the red and NIR bands, was incorporated to reduce the effects of illumination, topographic heterogeneity, and soil reflectance [80]. Processing in GEE included: (i) masking of clouds, shadows, and cirrus in each scene, generating a binary image that excluded affected pixels from the mosaic computation; (ii) rescaling of digital values to reflectance units; (iii) calculation of the median of unmasked pixels; and (iv) calculation of the NDVI and its stacking with the remaining bands.

### 4.5. Definition of Training Areas

Training areas were delineated through unsupervised classification using the K-means algorithm in Google Earth Engine (GEE), based on stacked image data and the spatial location of vegetation alliances identified through independent field surveys conducted prior to any spatial analysis. It is critical to note that K-means was applied exclusively as a spatial delineation tool—a computationally consistent alternative to manual polygon digitizing or fixed-radius buffers—and had no role in defining or influencing class labels, which were determined entirely by field-based phytosociological criteria. The procedure included: (i) pixel sampling according to geometry, scale, and required quantity; (ii) training of the algorithm with the number of vegetation alliances plus four general cover types (grassland, agricultural use, urban areas, and water bodies); (iii) classification of the multidimensional image into a single-band raster with cluster identifiers; and (iv) export at a spatial resolution of 30 m. Raster values not corresponding to natural forests were reclassified as “NoData” and generalized to a minimum mapping unit of 5 ha. The generalization process included: (i) thematic aggregation, grouping adjacent similar pixels using a focal majority filter applied to a 2 × 2 moving window; (ii) clump, identifying contiguous pixel groups per thematic class based on adjacency; and (iii) eliminate, iteratively replacing pixel groups below the 5 ha threshold with surrounding values meeting the required pixel count. The generalized thematic raster was subsequently vectorized, and polygons with spatial coincidence with vegetation sampling records—represented as point geometries—were retained as training areas [94]. While this delineation strategy implies that training pixels are spectrally coherent by construction—an inherent property of any segmentation-based approach, including visual interpretation—the independence of class labels from the clustering procedure ensures that the Random Forest classifier learns to discriminate phytosociologically defined units, not algorithmically generated groupings. This distinction is methodologically relevant and is acknowledged as a boundary condition of the approach.

### 4.6. Random Forest Classifier Configuration

**Figure 6:**
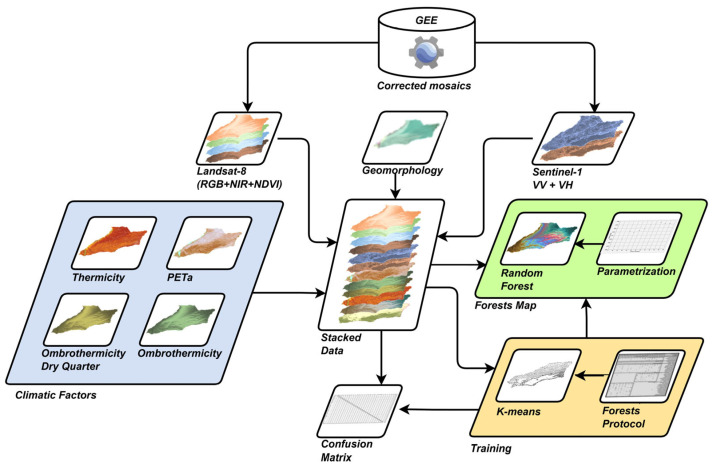
Methodological workflow for the spatial modeling of forests in the Colombian Orinoquia. The process integrates multi-source data and machine learning to classify and validate forest areas.

The configuration was based principally on the calculation of the optimal number of decision trees and training variables: (i) generation of samples from the multidimensional image and training polygons; (ii) partitioning of the dataset into 70% for prediction and 30% for validation, ensuring complete independence between subsets such that no training samples were included in the validation partition [44]; (iii) iteration with increasing numbers of trees—evaluated at intervals of 10 trees from 50 to 200—to estimate accuracy without overfitting, with the resulting accuracy curve used to identify the point of stability beyond which additional trees yielded no meaningful improvement [56,129]; (iv) configuration of the classifier with the optimal number of trees identified from this curve; (v) training with 70% of the data and execution with numerical attributes and stacked bands; and (vi) validation with the remaining 30%, calculation of the confusion matrix, and generation of accuracy curves by number of decision trees. The final classification, also executed in GEE, employed the optimal number of decision trees and the same training variables [58]. The result was exported as a raster at 30 m resolution, generalized to a minimum mapping unit of 5 ha, and vectorized. Confusion matrices, overall accuracy, producer and user accuracies, and the Kappa coefficient were calculated. Figure 6 presents the methodological synopsis employed to model the current distribution of forests in the Orinoquia.

## 5. Conclusions

The present study demonstrates that the integration of phytosociological field data, environmental complex variables, and multi-sensor satellite imagery within a Random Forest classification framework constitutes a robust and replicable approach for characterizing forest vegetation distribution at the regional scale. Nevertheless, the less spatially extensive formations—particularly *Phenakospermo guyannensis-Attaleetion maripae*, *Protio heptaphylli-Jacarandion obtusifoliae*, *Coccolobo caracasanae-Tapiriretion guianensis*, and *Gongylolepis martiana-Bonnetia sessilis*, collectively occupying less than 0.30% of the regional area—returned the highest omission errors (15.7–16.7%) and warrant prioritized ground validation efforts to refine classification boundaries and confirm the spatial extent of these formations. The 24 phytosociological alliances or geobotanical formations identified across the Colombian Orinoquia represent a significant advance over existing land cover surrogates, resolving the floristic and physiognomic complexity of approximately 7,565,696 ha of forest cover with high spatial accuracy and thematic detail.

The spatial organization of these formations is governed primarily by the geomorphological gradient—fluvial, denudational, and structural—and its associated edaphic−hydrological regimes, with mesoclimatic factors operating as complementary modulators of beta diversity across subregions. Anthropic transformation, driven predominantly by cattle ranching, agricultural expansion, and selective logging, constitutes the principal threat to forest cover, particularly in the Piedmont and Alluvial Plain subregions, where the most accessible and fertile environments have been most extensively altered. The persistence of forest remnants in areas of low agricultural suitability underscores both the passive protective role of topographic marginality and the urgent need for active conservation strategies in these refugia.

The cartographic framework produced here provides an empirically validated foundation for biodiversity assessments, ecosystem service valuation, habitat modeling, and ecological restoration planning in the Colombian Orinoquia. The methodological approach demonstrated is directly transferable to other subregions of the Orinoquia and to analogous Neotropical landscapes where detailed vegetation mapping remains a critical and unresolved challenge for conservation science and natural capital management.

## Figures and Tables

**Table 1:** Area of the Orinoquia currently covered by natural forests, and maximum omission and commission errors, based on Random Forest models.

Phytosociological Alliance or Geobotanical Formation	Area (ha)	Area (%)	OE Máx (%)	CE Máx (%)
*Bowdichio virgilioidis-Curatellion americanae*	541,913.67	2.32	5.4	7.6
*Gongylolepis martiana-Bonnetia sessilis*	25,732.04	0.11	15.7	13.2
*Alchorneo triplinerviae-Maurition flexuosae*	237,443.02	1.02	4.3	9.0
*Attaleo maripae-Iryantherion laevis*	3,478,544.43	14.89	1.8	2.3
*Batocarpo orinocensis-Senefelderion verticillatae*	101,250.31	0.43	12.2	7.9
*Brosimo lactescentis-Euterpion precatoriae*	113,448.85	0.49	11.4	4.0
*Chamaedoreo pinnatifrondis-Sloaneion brevispinae*	337,508.51	1.45	4.5	8.4
*Coccolobo caracasanae-Tapiriretion guianensis*	21,479.33	0.09	16.7	12.8
*Copaifero pubiflorae-Protion guianensis*	144,128.58	0.62	11.1	7.6
*Duguetio quitarensis-Amphirrhocion longifoliae*	310,583.23	1.33	7.3	3.3
*Enterolobio schomburgki-Terminalion amazoniae*	195,241.85	0.84	8.9	2.7
*Lueheo seemani-Pseudolmedion laevigatae*	31,621.22	0.14	14.4	10.3
*Guatterio hirsutae-Oenocarpion minoris*	227,847.09	0.98	8.8	4.0
*Micranda spruceana and species of Eschweilera*	284,104.91	1.22	5.2	4.7
*Ocoteo cernuae-Viticion orinocensis*	208,635.48	0.89	7.2	3.8
*Oenocarpo minoris-Attaleion maripae*	243,766.50	1.04	6.7	9.2
*Oenocarpus bataua-Protium rhoifolium*	172,849.21	0.74	9.5	6.9
*Phenakospermo guyannensis-Attaleetion maripae*	7518.72	0.03	16.1	11.8
*Protio aracouchini-Oenocarpion batauae*	45,944.29	0.20	13.6	9.2
*Protio guianensis-Caraipion llanori*	229,229.48	0.98	6.4	3.4
*Protio heptaphylli-Jacarandion obtusifoliae*	9484.38	0.04	16.3	10.5
*Siparuno guianensis-Maurition flexuosae*	221,191.47	0.95	10.5	7.1
*Spondiado mombinis-Viticion orinocensis*	424,117.03	1.82	6.1	8.6
*Syagro orinocensis-Virolion elongatae*	160,110.02	0.69	9.2	6.3

## Data Availability

The original contributions presented in this study are included in the article. Further inquiries can be directed to the corresponding author.
